# Higher locoregional recurrence rate for triple-negative breast cancer following neoadjuvant chemotherapy, surgery and radiotherapy

**DOI:** 10.1186/s40064-015-1116-2

**Published:** 2015-07-30

**Authors:** Chi Zhang, Shuang Wang, Hayley P Israel, Sherry X Yan, David P Horowitz, Seth Crockford, Daniela Gidea-Addeo, K S Clifford Chao, Kevin Kalinsky, Eileen P Connolly

**Affiliations:** Department of Radiation Oncology, New York Presbyterian Hospital, Columbia University Medical Center, New York, NY USA; Columbia University School of Medicine, New York, NY USA; Department of Medical Oncology, New York Presbyterian Hospital, Columbia University Medical Center, New York, NY USA; Department of Biostatistics, Columbia University School of Medicine, New York, NY USA

**Keywords:** Triple-negative breast cancer, Locoregional recurrence rate, Neoadjuvant chemotherapy, Radiation

## Abstract

**Background:**

Breast cancer subtype, determined by expression of estrogen/progesterone receptor (ER/PR) and human epidermal growth factor receptor (HER)-2, is predictive for prognosis. The importance of subtype to locoregional recurrence (LRR) following neoadjuvant chemotherapy (NAC) is unknown, particularly after adjuvant radiotherapy (RT).

**Methods:**

We retrospectively identified 160-breast cancer patients registered at Columbia University Medical Center from 1999 to 2012 treated with NAC, surgery and adjuvant RT.

**Results:**

Patients were grouped by receptor status: hormone receptor positive (HR+) [(ER or PR+)/HER2−; n = 75], HER2+ (n = 46), or triple-negative (TNBC) [ER (−) PR (−) HER2 (−); n = 36]. The median follow-up was 28 months. 92.0% received an anthracycline-taxane based NAC and 80.4% of HER2+ patients received trastuzumab. All underwent surgical resection followed by RT. 15.6% had a pathologic complete response (pCR): 26% of HER2+, 5% of HR+, and 25% of TN. The actuarial rate of DM was 13.8% for the entire cohort, with equivalent rates by subtypes in non-pCR patients. The overall rate of LRR was 8%. However, the LRR rate was significantly higher for TNBC patients (22.2%) than HER2+ (5.6%) (p = 0.025) or HR+ (3.0%) (p = 0.037) in non-pCR group. In the pCR group, two patients had recurrence; one LRR and one a DM, both had TNBC. All LRR occurred in or near the radiation field.

**Conclusions:**

TNBC patients with < pCR to NAC have a significantly higher LRR rate as compared to other subtypes even with surgery and adjuvant RT. Our data support a need to further intensify local therapy in TNBC patients.

## Background

Triple-negative breast cancers (TNBC), i.e., tumors negative for ER, PR and HER2, comprise 15–20% of breast cancers in the United States but account for a disproportionate share of morbidity and mortality (Boyle 2012). Gene expression studies using microarrays have identified four common subtypes of breast cancer that are not apparent using traditional histopathologic methods: luminal A, luminal B, HER2 positive, and basal-type (Perou et al. 2000; Sorlie et al. 2001, 2003). Most TNBCs are high-grade invasive ductal carcinomas, approximately 80% of which display a basal-like pattern when analyzed by gene expression (mRNA) microarray (Rakha et al. 2008; Lehmann et al. 2011). A fraction (>60%) of tumors with borderline ER expression (1 to 10%) is also classified as basal-like (Deyarmin et al. 2013).

TNBC predicts for poorer overall survival (OS) and distant metastasis (DM) following treatment (Abdulkarim et al. 2011; Haffty et al. 2006; Nguyen et al. 2008; Dent et al. 2007). Whether TNBC is associated with higher rates of locoregional recurrence (LRR) is controversial, previous studies combined patients with or without adjuvant RT and reported rates of LRR from 4 to 29% (Abdulkarim et al. 2011; Haffty et al. 2006; Nguyen et al. 2008; Dent et al. 2007; Millar et al. 2009; Dominici et al. 2012; Liedtke et al. 2008). Neoadjuvant chemotherapy (NAC) is increasingly being used in women with locally advanced breast cancer or high-risk early stage disease (White and Mamounas 2014). Numerous reports on the chemosensitivity, and prognosis of different subtypes of breast cancers to NAC have been published with poorer overall outcome again observed in TNBCs (Rouzier et al. 2005; Meyers et al. 2011; Esserman et al. 2012; Rastogi et al. 2008; Mieog et al. 2007; von Minckwitz et al. 2012; Berry et al. 2006; Hugh et al. 2009; Saigal et al. 2013; Caudle et al. 2012; Carey et al. 2007; Nielsen et al. 2004). Nonetheless, the correlation between subtypes of breast cancer and rates of LRR in the setting of NAC is not well characterized. We thus have conducted a single institutional retrospective study comparing the LRR rate of TNBCs with other subtypes of breast cancer for patients treated with trimodality therapy: NAC, surgery and all with adjuvant RT.

## Methods

### End points and statistical methods

LRR was defined as first site of failure being locoregional to primary site, which includes the chest wall/intact breast, ipsilateral axilla, internal mammary nodes, or SCV fossa. Progression-free survival (PFS) was defined as the time from the date of surgery to earliest occurrence of LRR or DM. Overall survival (OS) was defined as the time from diagnosis to date of death due to any cause. A pCR to NAC was defined as no residual invasive disease in the breast or axilla at surgery.

All statistical analyses were conducted with SPSS software (IBM SPSS 21) and SAS software 9.2. Rates of LRR-free, progression-free, and overall survival (OS) were calculated by the Kaplan–Meier method, with comparisons among groups performed with log-rank tests. Cox regression analyses were used for multivariate analysis. Chi-squared or Fisher’s exact tests were used for analysis with categorical variables. All P values were two-sided with α = 0.05.

### Patient population

This study was conducted under an institutional review board approved protocol (IRB-AAAJ8512) at Columbia University Medical Center. A waiver for consent to participate was granted for all patients involved by the IRB for this retrospective studies. Under this approved protocol (IRB-AAAJ8512), we retrospectively identified all breast cancer patients registered at Radiation Oncology at CUMC from 1999 to 2012. Only patients who were treated with NAC, surgery and adjuvant RT were included.

### Breast cancer subtype classification

Basal-like subtype was largely hormonal receptor negative (HR−) (ER−/PR−and HER2−, the HER2 enriched subtype was HR− and HER2+, and the luminal subtype (luminal A and B combined) was ER+ (Carey et al. 2007; Nielsen et al. 2004). For this study, three subtypes are defined: (1) Hormonal receptor positive (HR+) (ER+ and/or PR+), and HER2/neu non-amplified (HER2−) tumors representing luminal types (luminal A and the majority of luminal B); (2) HER2+ tumors representing HER2 enriched and a minority of luminal B; (3) Triple negative (ER−, PR−, and HER2−) breast cancers representing basal-like tumors. Tumors were considered HER2+ if they were 3+ by IHC or positive for HER2/neu gene amplification by fluorescence in situ hybridization.

## Results

### Demographics

Table 1 illustrates the patient characteristics and the treatments of the tumors applied. 160 patients were treated with NAC, surgery and adjuvant RT diagnosed between year 1999 and 2012. 75 patients had HR+/HER2− tumors, 46 patients had HER2+ tumors and 36 patients had TNBCs. Three patients had no HER2 status reported thus were not grouped into either of the subtypes. Of the 46 patients with HER2+ disease, 9 were HR+/HER2+ with the rest HR−/HER2+. Median age at diagnosis was 52 years, not significantly different among the three subtypes. Our cohort had a high percentage of black and Hispanic patients, all together 44.4% (16.9 and 27.5% respectively). The percentage was even higher for TNBCs, 58.4% black and Hispanic (27.8 and 30.6% respectively).

**Table 1:** Demographics and tumor characteristics

Variables	Total		HR+/HER2−		HER2+		TN		P
Age at diagnosis, median (range)	52 years (25–85)		52 (26–85)		50 (27–73)		50 (31–84)		
≤50 years	75	(46.9%)	33	(44.0%)	23	(50.0%)	19	(52.8%)	0.64
>50 years	85	(53.1%)	42	(56.0%)	23	(50.0%)	17	47.2%)	
Race/ethnicity									
White, non-Hispanic	59	(36.9%)	29	(38.7%)	19	(41.3%)	9	(25.0%)	0.29
Black, non-Hispanic	27	(16.9%)	10	(13.3%)	7	(15.2%)	10	(27.8%)	
Hispanic	44	(27.5%)	18	(24.0%)	14	(30.4%)	11	(30.6%)	
Other or unknown	30	(18.8%)	18	(24.0%)	6	(13.0%)	6	(16.7%)	
Laterality									
Left	80	(50.0%)	42	(56.0%)	20	(43.4%)	17	(47.2%)	0.42
Right	79	(49.4%)	33	(44.0%)	25	(54.3%)	19	(52.8%)	
Bilateral	1	(0.6%)	0	(0%)	1	(2.2%)	0	(0%)	
Pathology									
IDC	128	(80.0%)	52	(69.3%)	40	(87.0%)	33	(91.7%)	0.02
ILC	15	(9.4%)	13	(17.3%)	1	(2.2%)	1	(2.8%)	
Mixed	14	(8.8%)	8	(10.7%)	5	(10.9%)	1	(2.8%)	
Other	3	(1.9%)	2	(2.7%)	0	(0%)	1	(2.8%)	
Chemotherapy									
chemo (AC/T backbone)	100	(62.5%)	46	(61.3%)	25	(54.3%)	28	(77.8%)	0.04*
non-AC/T backbone	50	(31.2%)	23	(30.7%)	20	(43.4%)	6	(16.7%)	
Unspecified	10	(6.2%)	6	(8.0%)	1	(2.2%)	2	(5.6%)	
Trastuzumab received									
	37 of 46	(80.4%)	N/A		37 of 46	(80.4%)	N/A		N/A
Surgery									
Mastectomy	114	(71.3%)	59	(78.7%)	32	(69.6%)	22	(61.1%)	0.23
Lumpectomy	44	(27.5%)	15	(20.0%)	14	(30.4%)	14	(38.9%)	
ALND only^a^	2	(1.3%)	1	(1.3%)	0	(0%)	0	(0%)	
Radiation									
Breast only (2 fields)	19	(11.9%)	7	(9.3%)	6	(13.0%)	6	(16.7%)	0.51
CW/breast + axillary + SCV LNs (3 or 4 fields)	112	(70.0%)	52	(69.3%)	32	(69.6%)	25	(69.4%)	
CW/breast + axillary + SCV + IMN (deep tangents or 5 fields or IMRT)	13	(8.1%)	5	(6.7%)	4	(8.7%)	4	(11.1%)	
Unspecified	16	(10.0%)	11	(14.7%)	4	(8.7%)	1	(2.8%)	
Boost to tumor cavity and/or scar	91	(56.9%)	43	(57.3%)	21	(45.6%)	27	(75.0%)	0.03
Stage (clinical)									
I	0	(0%)	0	(0%)	0	(0%)	0	(0%)	0.46*
II	78	(48.8%)	38	(50.7%)	19	(41.3%)	20	(55.6%)	
III	71	(44.3%)	31	(41.3%)	23	(50.0%)	15	(41.7%)	
IV^b^	3	(1.9%)	1	(1.3%)	2	(4.3%)	0	(0%)	
Unknown	8	(5.0%)	5	(6.7%)	2	(4.3%)	1	(2.8%)	
Stage (pathologic)									
pCR (total)	25	(15.6%)	4	(5.3%)	12	(26.1%)	9	(25.0%)	0.001
T0	19	(11.9%)	4	(5.3%)	8	(17.4%)	6	(16.7%)	
Tis	6	(3.7%)	0	(0%)	4	(8.7%)	3	(8.3%)	
I	25	(15.6%)	8	(10.7%)	10	(21.7%)	7	(19.4%)	0.07*
II	49	(30.6%)	26	(34.7%)	10	(21.7%)	12	(33.3%)	
III	59	(36.9%)	37	(49.3%)	12	(26.1%)	8	(22.2%)	
IV	1	(0.6%)	0	(0%)	1	(2.2%)	0	(0%)	
Unknown	1	(0.6%)	0	(0%)	1	(2.2%)	0	(0%)	

* P values were calculated with the unknown or unspecified group excluded. All P-values were calculated using Pearson’s Chi-squared tests.

^a^Two patients had only axillary nodal dissection due to no clinical evidence of primary cancer found in the breast.

^b^Three patients presented with likely stage IV disease with one patient having lung nodules on CT scan, one with adrenal uptake and the other patient with sternal uptake on PET scan, all resolved after NAC, thus were treated definitively afterward.

62.5% of the cohort received Adriamycin/Cyclophosphamide/Taxol (AC/T) as NAC while 31.2% had AC only, T only, CMF or other types. Patients with TNBCs had a significantly higher rate (77.8%) of receiving AC/T backboned NAC than other subtypes (p = 0.04). 80.4% of HER2+ patients received Trastuzumab. 27.5% patients had lumpectomy following NAC. 71.3% had modified radical mastectomy including 16 cases of skin or nipple sparing mastectomy.

All patients received RT, for 144 patients (90%) all details of RT setups are known. All patients had radiation to chest wall after mastectomy or the whole breast after lumpectomy. 78.1% patients received regional nodal irradiation covering axillary and supraclavicular nodes using three- or four-field technique, including 8.1% of patients with internal mammary nodes irradiated. TNBC patients had a higher rate (75%) of receiving boost RT to scar or tumor cavity than the other subtypes (p = 0.03). The majority of patients were treated using 3D conformal radiation techniques with only 5% treated with inverse planning intensity modulated radiation therapy (IMRT).

### Response to NAC

A majority of the patients presented with clinical stage II and III cancer (Table 1). Down staging of the disease was observed after NAC. 25 patients (15.6%) achieved pCR with no invasive disease identified from surgery in primary breast or axilla. HR+/HER2− patients had significant lower ratio of achieving pCR (5.3%) comparing with HER2 + (26.1%) (p = 0.001) and TNBCs (25%) (p = 0.003). Another 25 (15.6%) patients had pathologic stage I disease after NAC, again with a much lower rate (about half) in HR +/HER2- subtype comparing with the other two subtypes.

### Treatment outcomes and survivals

After a median follow-up of 28 months (5 months–13.7 years), the 3-year OS was 85.4%; the 3-year progression-free survival (PFS) was 68.5%. The 3-year OS was 100% for patients who achieved pCR after NAC compared to 82.7% for patients without pCR (p = 0.06). The 3-year PFS was 87.5% for pCR vs. 65.2% for non-pCR patients (p = 0.08). Only one patient (cT2 cN1 M0) with pCR had LRR that occurred 26 months after finishing RT. Another patient (cT2 cN3 M0) with pCR had DM to the brain 2 months after RT. Both had TNBC, received AC/T followed by lumpectomy and axillary lymph node dissection. The patient with LRR had RT to the whole breast while the patient with DM received RT to whole breast, full axillary and supraclavicular nodes. Patients with non-pCR had poorer prognosis with a total of 11 LRRs including two patients with concurrent LRR and DM, and 19 patients with DM as the first site of failure. For the entire cohort, LRRs occurred within 29 months after surgery (median time to LRR was 13 months).

In the overall cohort, significantly higher rate of LRR was shown in TNBCs (19.4%) compared to HR+/HER2− (5.33%) (p = 0.037) and HER2+ (2.18%) (p = 0.019) (Table 2). The difference was even more prominent for non-pCR patients. LRR rate in TNBCs was 22.2 vs. 5.6% in HR+/HER2− (*p* = 0.025) and 2.9% in HER2+ groups (p = 0.037). Interestingly, no significant difference was found in DM among the subtypes as the first site of failure (p = 0.73).

**Table 2:** Incidences of LRR, DM and death from all causes among different subtypes

Events	Incidences		Subtypes							P**
TNBC is defined as ER/PR positive staining in <1% cells										
	All^a^ (n = 157)		HR+/HER2− (ER/PR ≥1%) (n = 75)			HER2+ (n = 46)		TN (ER/PR <1%) (n = 36)		P
LRR	12^b^	(7.6%)	4		(5.3%) (p = 0.037)^π^	1	(2.2%) (p = 0.019)^π^	7	(19.4%)	0.014
DM	22	(14.0%)	11		(14.7%) (p = 0.78)^π^	5	(10.9%) (p = 0.52)^π^	6	(16.7%)	0.73
Death	21	(13.4%)	8		(10.7%) (p = 1.0)^π^	7	(15.2%) (p = 0.75)^π^	4	(11.1%)	0.77
	Non-pCR (n = 132)		HR+/HER2− (ER/PR ≥1%) (n = 71)			HER2+ (n = 34)		TN (ER/PR <1%) (n = 27)		P
LRR	11	(8.3%)	4		(5.6%) (p = 0.025)^π^	1	(3.0%) (p = 0.037)^π^	6	(22.2%)	0.023
DM	21	(15.9%)	11		(15.5%) (p = 0.76)^π^	5	(14.7%) (p = 0.74)^π^	5	(18.5%)	0.90
Death	19	(14.4%)	8		(11.3%) (p = 0.73)^π^	7	(20.6%) (p = 0.74)^π^	4	(14.8%)	0.40
TNBC is defined as ER/PR positive staining in <10% cells										
	All^a^ (n = 157)		HR+/HER2− (ER/PR ≥10%) (n = 68)			HER2+ (n = 46)		TN (ER/PR <10%) (n = 43)		P
LRR	12^b^	(7.6%)	2	(2.9%) (p = 0.003)^π^		1	(2.2%) (p = 0.006)^π^	9	(20.9%)	0.001
DM	22	(14.0%)	9	(13.2%) (p = 0.59)^π^		5	(10.9%) (p = 0.37)^π^	8	(18.6%)	0.59
Death	21	(13.4%)	5	(7.3%) (p = 0.21)^π^		7	(15.2%) (p = 1.0)^π^	7	(16.3%)	0.27
	Non-pCR (n = 132)		HR+/HER2− (ER/PR ≥10%) (n = 64)			HER2+ (n = 34)		TN (ER/PR< 10%) (n = 34)		P
LRR	11	(8.3%)	2	(3.3%) (p = 0.003)^π^		1	(2.9%) (p = 0.027)^π^	8	(23.5%)	0.002
DM	21	(15.9%)	9	(14.8%) (p = 0.41)^π^		5	(14.7%) (p = 0.75)^π^	7	(20.6%)	0.68
Death	19	(14.4%)	5	(7.8%) (p = 0.10)^π^		7	(20.6%) (p = 1.0)^π^	7	(20.6%)	0.11

Triple negative breast cancer was defined as ER and PR positive staining either in <1% cells or in <10% cells as shown respectively.

All LRR and DM are counted as first site of failure.

** P values are obtained using Fisher’s exact tests with two degree-of-freedom.

^╥^P values are calculated using Fisher’s exact tests comparing with TN subtype.

^a^There were three patients who had no HER2 status documented.

^b^Two of the LRR occurred simultaneously with DM.

The above analyses were conducted by defining HR− as both ER <1% and PR <1% based on the current standard (Hammond et al. 2010). Due to reports that weakly ER/PR+ disease harbors a significant portion of basal-like tumors (Deyarmin et al. 2013), we reanalyzed the LRR rates redefining TNBC subtype as being both ER and PR <10% and HER2− (Table 2). The results showed two additional cases of LRR in the TNBCs (<10%) as additional 7 cases (originally in HR+/HER2− group) were included in the redefined TNBC subtype. The redefined TNBCs (<10%) had even higher rate (23.5%) of LRR in non-pCR patients when comparing to HR+/HER2− (3.3%) (p = 0.003) and HER2+ subtypes (2.9%) (p = 0.027). In non-pCR group, the highest rate of DM was seen in TNBCs (20.6%), compared with HR+/HER2− (14.8%) and HER2+ subtypes (14.7%) but the difference did not reach statistical significance (p = 0.41 and p = 0.75, respectively). No statistically significant difference was found among the subtypes of the crude death rate for the entire cohort or non-pCR patients by definition of HR <1% or <10%.

We reviewed the radiation field setup and individual RT plans delivered to all patients with LRR. The pattern of failure was summarized in Table 3. All LRRs occurred either in or near radiation fields. The only LRR in the pCR group occurred 28 months after lumpectomy and whole breast RT (5,000 cGy followed by 1,000 cGy boost to tumor bed). The patient recurred with a skin nodule in the inframammary fold within the RT field but not near the lumpectomy margin. Of the 11 LRRs in non-pCR patients, seven occurred in the intact breasts (after lumpectomy) or chest wall (after mastectomy) and four of them occurred in the irradiated regional nodal area.

**Table 3:** Pattern of failure and locations of LRR

	LRR			DM
	In-field	Near-field	Out-field	
pCR	1	0	0	1^a^
Non-PCR	10	1	0	21^b^

^a^This patient had brain metastasis 2 months after RT (5 months after surgery).

^b^Lung metastases 9, brain 6, bone 4, Liver 4, contralateral axilla 1, retrosternal 1. A few patients had simultaneous DM in multiple organs.

**Fig. 1:**
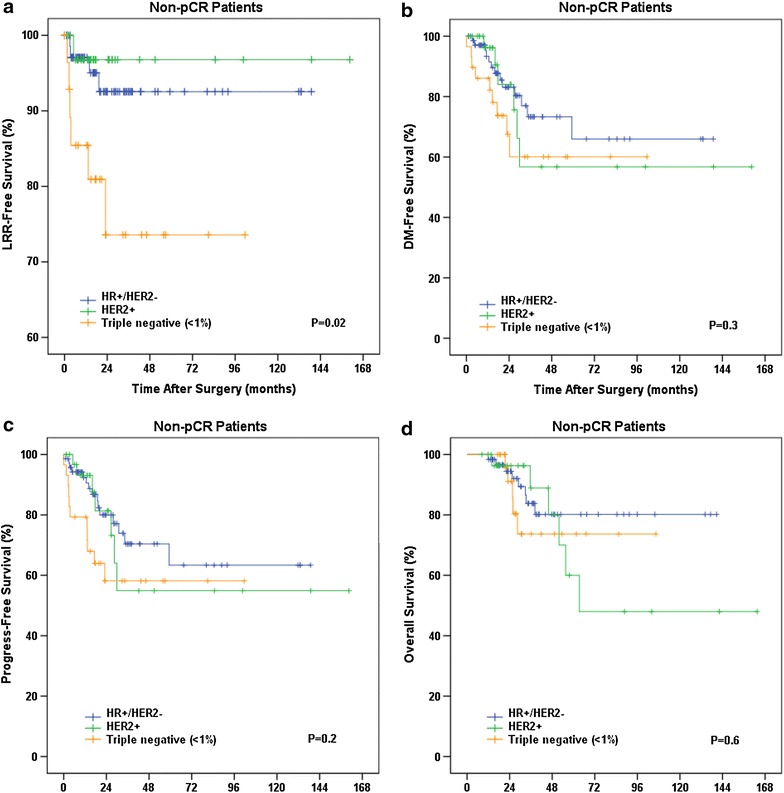
Comparisons of survivals between subtypes in non-pCR patients. **a** Locoregional-recurrence-free survival (LRR-Free Survival). **b** Distant-metastasis-free survival (DM-Free Survival). **c** Progress-free survival. **d** Overall survival. P values shown here are from comparisons among all three subtypes.

Using Kaplan–Meier analyses, we have further compared the LRR-free, DM-free, Progression-free and overall survivals among different subtypes in non-pCR patients (Fig. 1). Log-rank studies showed significantly higher risk of LRR in TNBCs (HR <1%) comparing to HR+/HER2− (p = 0.033) and HER2+ subtypes (p = 0.02). DM-free survival curve showed relatively early onset of DM in TNBCs but the difference was not significant among subtypes (p = 0.6). PFS again did not show statistically significant difference among subtypes (p = 0.2) likely due to similar DM rates in different subtypes. Median OS time has not been reached in our study. Although the 3- or 5-year OS rates among different subtypes are not different significantly, it is noted that HER2+ patients have relatively later occurrence of death.

14 patients were identified among the 160 in our database with inflammatory breast cancer (IBC); two with stage IV and the remaining stage III at diagnosis. 8/14 have HER2+ disease, 5/14 are HR+ (>1%)/HER2-, and one patient with TNBC (HR <1%). 13/14 had modified radical mastectomy, one patient had no ALND performed due to chest wall and nerve involvement of the tumor and eventually had local recurrence in chest wall. All patients received adjuvant RT to chest wall and axilla (ranging from 5,000 to 5,040 cGy) and SCV (4,500–5,040 cGy), with seven patients receiving boost dose of RT to the scar (1,000–2,000 cGy). Three patients had LRR, including the patient with TNBC; the other two had HR+ (<10%)/HER2− and HER2+ disease. Five patients had DM as first relapse, three with HR +/HER2- and two with HER2+ disease. Seven patients died over a median of 29 months after diagnosis (range 11.9–55.6).

### Risk factors for LRR

Univariate analysis (UVA) of the patients’ and tumor characteristics showed the following factors predicting higher risk of LRR: subtype (p = 0.019), race/ethnicity (p = 0.014), surgical pathologic grade (post-neoadjuvant chemotherapy) (p = 0.005) and lymphovascular invasion (LVI) (p = 0.021). Age (≤50 vs. >50 years) (p = 0.068), clinical staging (p = 0.24), pathologic stage (p = 0.31), mitotic index (p = 0.29), type of chemotherapy (p = 0.75), margin status (p = 1.0) and grade of tumor from biopsy (pre-NAC) (p = 0.34) did not show significant correlations with higher risks of LRR per UVA. Multivariate analysis (MVA) confirmed that TNBCs were associated with significantly higher risk of LRR with hazard ratio (HR) being 3.33 compared with non-TNBCs (95% confidence interval (CI) 1.04–10.70, p = 0.043) (Table 4). With LRR and DM included in PFS calculations, TNBCs was again seen with worse PFS (HR 1.94, CI 0.92–4.09) but results from MVA were not statistically significant (p = 0.083). There was a significantly higher risk of LRR (p = 0.014) and progression (p = 0.001) in patients with high grade of tumor at surgery in MVA.

**Table 4:** Cox regression analyses on LRR and PFS

MVA variants	LRR		PFS	
	Hazard ratio (95%CI)	P	Hazard ratio (95%CI)	P
TN vs. not TN	3.33 (1.04–10.70)	0.043	1.94 (0.92–4.09)	0.083
Surgical pathologic grade				
Grade 3 vs. other^a^	6.93 (1.48–32.53)	0.014	3.70 (1.66–8.22)	0.001
Age >50 vs. ≤50 years	0.46 (0.12–1.77)	0.26	0.91 (0.43–1.94)	0.81

^a^This includes grade 1, 2 and the patients who had pCR after NAC.

## Discussion

Numerous studies have reported that TNBC is associated with a poor prognosis. Recently, more attention has been paid to patients receiving neoadjuvant chemotherapy (NAC). Whether the rate of LRR in certain subtypes of breast cancer is higher, particularly in TNBCs, is unknown, partly due to the heterogeneity of patients and their treatment regimens reported in prior studies (Abdulkarim et al. 2011; Haffty et al. 2006; Nguyen et al. 2008; Dent et al. 2007; Millar et al. 2009; Dominici et al. 2012; Liedtke et al. 2008; Rouzier et al. 2005; Panoff et al. 2011; Bauer et al. 2007). In our patient cohort comprised mainly of clinical stage IIb to III disease, every patient received a course of NAC, surgery and radiation. About two-thirds of the patients (62.5%) received AC/T backboned NAC. The majority of patients had mastectomy (71.3%). 78.1% patients received extensive RT covering chest wall or intact breast, axillary and supraclavicular nodes.

Our retrospective study revealed that TNBCs are associated with significantly increased LRR rate comparing to other subtypes. Our data showed an accumulated incidence of 19.2% LRR in TNBCs, much higher than other subtypes (4.1% in non-TNBC group). Results were significant by UVA and MVA. All LRRs occurred within 28 months after surgery (median time to LRR in TNBC group is 3.6 vs. 5 months in non-TNBCs, p > 0.05). The overall DM (first site of failure) rates were similar among subtypes. The LRR rate of TNBCs in our patient population is slightly higher compared to the other studies. For example, the studies from Caudle et al. showed 10.5% 5-year LRR in TNBCs after NAC, lumpectomy and adjuvant RT while only 3% in luminal A type (Caudle et al. 2012). The relatively higher percentage of lower clinical stages at presentation (71% clinical stage I/II disease) in that study may explain the low incidence of LRR. Another study from University of North Carolina reported an overall 14% LRR rate of TNBCs but only 4% in non-TNBCs with a median follow up of 55 months (Meyers et al. 2011). However, not all patients in these studies received adjuvant RT.

Another explanation for the higher incidence of LRR in our data may be the significantly higher proportion of non-White patients in our cohort, which is not seen in other studies (Meyers et al. 2011; Caudle et al. 2012). 44.4% patients in our study were non-White, including black non-Hispanic (16.9%) and Hispanic (27.5%), while 36.9% are White non-Hispanic. The study from Meyers et al. (2011) included a much higher proportion of White patients (62%). Patients with TNBCs are significantly more likely to be African Americans and Hispanics and this is reported to confer worse prognosis, including mortality (Deyarmin et al. 2013; Carey et al. 2006; Lund et al. 2009; Vona-Davis and Rose 2009; Maskarinec et al. 2011; Wu et al. 2013). We have confirmed this finding by showing a significant difference of cumulative mortality in our Hispanic (27.3%), black non-Hispanic (14.8%) and white non-Hispanic group (6.8%) (p = 0.02) (Table 5). In addition, a significantly higher cumulative incidence of LRR was seen in black non-Hispanic patients (22.2%) comparing to white non-Hispanics (p = 0.02), correlating with the higher rate of mortality. This was not observed in Hispanic patients, LRR rate 6.8 vs. 5.1% in White non-Hispanics (p = 1.0). The LRR reached an exceptionally high rate of 40% in black non-Hispanic patients with TNBCs, with four incidences out of a total of ten patients. The LRR rate in Hispanic patients with TNBCs was 18%, with two incidences out of a total of 11 patients. The LRR rate in White non-Hispanics was 11%, with one LRR out of a total of nine patients. Hispanic patients showed a somewhat higher rate of DM than the other groups but the difference was not statistically significant. Overall, our data suggested that both black and Hispanic patients had worse prognosis in survival than White non-Hispanic patients, consistent to previous reports. We have, for the first time, reported the significantly increased risk of LRR in black non-Hispanic patients, particularly high in black non-Hispanics with TNBCs (40% LRR).

**Table 5:** Incidences of LRR, DM and death among different races/ethnicities

Events	All Cohort (160)		White non-Hispanic (59)		Black non-Hispanic (27)		Hispanic (44)		Other and unknown^a^ (30)		P**
LRR	12	(7.5%)	3	(5.1%)	6	(22.2%) (p = 0.02)^╥^	3	(6.8%) (p = 1.0)^╥^	0	(0%)	0.04
DM	22	(13.8%)	8	(13.6%)	3	(11.1%) (p = 1.0)^╥^	10	(22.7%) (p = 0.30)^╥^	1	(3.3%)	0.39
Death	21	(13.1%)	4	(6.8%)	4	(14.8%) (p = 0.25)^╥^	12	(27.3%) (p = 0.006)^╥^	1	(3.3%)	0.02

** P values are calculated via Fisher’s exact tests, two degree-of-freedom, without the “other and unknown” group.

^╥^P values are calculated using Fisher’s exact tests comparing with White non-Hispanic.

^a^Other and unknown: 24 patients self-identified as other, three are Asians, and three are unknown.

Deyarmin et al. recently showed that low-ER-staining (1–10%) tumors were clinicopathologically more similar to ER-negative than to ER-positive tumors (Deyarmin et al. 2013). Using molecular subtyping, 62% of the low-staining tumors were classified as basal like and 27% as HER2 enriched. In our study, the rate of LRR in TNBC group was more prominent if using the definition of hormonal receptor positivity being ≥10% of stained cells instead of the ASCO/CAP guideline of being ≥1%. The crude relative risks of LRR of TNBCs vs. non-TNBCs increased from 4.7 (HR <1%) to 7.9 (HR <10%). Our data may serve as an indirect evidence to support the claim of Deyarmin et al. that “10%” could be a more accurate threshold to define ER-positivity.

Further followup of patients with LRR revealed that local failure led to high risk of DM and cancer-specific death. Of the ten patients with LRR as the only site of first failure, eight developed DM with a median time interval of 7 months after LRR (range 1–12 months). One patient was lost to follow up after treatment and the other one had a suspicious bony metastasis but lost to follow up before DM was confirmed. Two of the eight patients (25%) died shortly after DM was diagnosed (both within 1 month). Our results indicate the importance of the locoregional control for improving overall prognosis.

After NAC, 15.6% patients overall achieved pCR. Among them, 25% TNBCs had pCR while only 5.3% in HR+/HER2− group had pCR, consistent to other reports that luminal A responded less to NAC comparing to other subtypes (Meyers et al. 2011; Rastogi et al. 2008; Mieog et al. 2007). In TNBCs, higher percentage (38.9%) received lumpectomy likely due to better response to NAC. However, we do not think that lumpectomy contributes to higher rate of LRR. Only one patient out of a total of 14 after lumpectomy developed LRR, while six LRR (3 locally, 2 locoregionally and 1 in regional nodes) occurred out of 23 TNBCs after mastectomy (p = 0.22). Our results indicated that lumpectomy is a valid surgical approach for TNBC patients without risks of increasing LRR in selected patients with good responses to NAC.

## Conclusion

In conclusion our study is the first to report an increased risk for LRR in TNBC patients following NAC in a cohort in which all patients received adjuvant RT. The risk of LRR is particularly high in African Americans with TNBC. The majority of patients received RT to chest wall/breast and regional lymph nodes, with more than half (including the majority of TNBC patients) receiving an additional boost dose of RT to the tumor bed and/or surgical scar. In spite of adjuvant RT, the incidence of LRR in TNBCs remains high. TNBC are reported to have an enriched content of cancer stem cells, which likely convey higher radioresistance (Atkinson et al. 2013), this may explain the high rate of LRR in those with <pCR. Given the importance of locoregional control in preventing future DM, our data indicate the need for intensification of therapy in TNBCs with <pCR to NAC. Options include adding radiosensitizing agents during radiation course and/or additional adjuvant chemotherapy after surgery. Identification of potential resistant subgroups of TNBCs to RT will also shed light on how to pre-select patients for treatment intensification.

## Abbreviations

TNBC: triple-negative breast cancer
ER: estrogen receptor
PR: progesterone receptor
HER-2: human epidermal growth factor receptor-2
NAC: neoadjuvant chemotherapy
pCR: pathologic complete response
RT: radiation therapy
LRR: locoregional recurrence
DM: distant metastasis
